# Protective ventilation reduces bacterial growth and lung injury in a porcine pneumonia model

**DOI:** 10.1186/cc13465

**Published:** 2014-03-17

**Authors:** J Sperber, A Nyberg, M Lipcsey, A Larsson, J Sjolin, M Castegren

**Affiliations:** 1 Centre for Clinical Research Sormland, Uppsala University, Uppsala, Sweden; 2 Uppsala University, Uppsala, Sweden

## Introduction

Bacterial pneumonia is a common indication for mechanical ventilation in the ICU. Ventilation with high positive end- expiratory pressure (PEEP) and low tidal volume (VT) is recommended in patients with adult respiratory distress syndrome. This improves clinical outcome compared with ventilation with low PEEP and medium-high VT [1]. However, the effect of VT and PEEP on bacterial growth in lung tissue is not known. This study contrasted the effect of a protective ventilator protocol with a standard medium-high VT and lower PEEP protocol on lung bacterial growth, lung edema formation and lung injury. It was performed in a porcine model of intensive care with the frequently found pathogen Pseudomonas aeruginosa.

## Methods

Sixteen pigs were anesthetized and randomized to mechanical ventilation with two different ventilator settings for 6 hours; Prot-V (PEEP 10 cmH_2_O, VT 6 ml/kg, *n *= 8) and Control (PEEP 5 cmH_2_O, VT 10 ml/kg, *n *= 8). At 0 hours, 1 × 10^11 ^colony-forming units (cfu) of P. aeruginosa were instilled intratracheally. At the end of the experiment, six postmortem lung biopsies from predefined declivial locations were taken from each animal for cultures and weight measurements.

## Results

P. aeruginosa growth in lung tissue and wet to dry ratio were lower in the Prot-V group than in the Control group (*P *< 0.05 and *P *< 0.05). PaO_2_/FiO_2 _was higher in the Prot-V group than in the Control group (*P *< 0.05) (Table 1).

**Table 1:** P. aeruginosa log mean,wet to dry ratio, and PaO_2_/ FiO_2_at 5 hours

	*P. aeruginosa *(cfu/g)	W/D	PaO_2_**/**/FiO_2 _at 5 hours (mmHg)
Prot-V	3.5 ± 0.7	1.7 ± 0.2	434 ± 62
Control	4.2 ± 0.7	2.7 ± 1.3	343 ± 61

Data presented as mean ± SD.

## Conclusion

Protective ventilation with low VT and higher PEEP reduces P. aeruginosa growth in lung tissue, lung edema formation and lung injury in contrast with medium-high VT and lower PEEP ventilation in this porcine pneumonia model.

## References

[B1] N Engl J Med 2000 342 1301 1308 10793162 10.1056/NEJM200005043421801

